# Multi-disciplinary Approach for Drug and Gene Delivery Systems to the Brain

**DOI:** 10.1208/s12249-021-02144-1

**Published:** 2021-12-03

**Authors:** Nkafu Bechem Ndemazie, Andriana Inkoom, Ellis Fualefeh Morfaw, Taylor Smith, Monica Aghimien, Dexter Ebesoh, Edward Agyare

**Affiliations:** 1 College of Pharmacy and Pharmaceutical Sciences, Florida A&M University, 1415 South Martin Luther King Blvd, Tallahassee, FL USA; 2 Foumban District Hospital, West Regional Directorate of Health, Bafoussam, West Region Cameroon; 3 College of Science and Technology, Florida A&M University, Tallahassee, FL USA; 4 ED Medical Illustrations, New York City, NY USA

**Keywords:** Drug delivery, Blood–brain barrier, Permeability, Nanoparticle delivery system

## Abstract

Drug delivery into the brain has for long been a huge challenge as the blood–brain barrier (BBB) offers great resistance to entry of foreign substances (with drugs inclusive) into the brain. This barrier in healthy individuals is protective to the brain, disallowing noxious substances present in the blood to get to the brain while allowing for the exchange of small molecules into the brain by diffusion. However, BBB is disrupted under certain disease conditions, such as cerebrovascular diseases including acute ischemic stroke and intracerebral hemorrhage, and neurodegenerative disorders including multiple sclerosis (MS), Alzheimer’s disease (AD), Parkinson’s disease (PD), and cancers. This review aims to provide a broad overview of present-day strategies for brain drug delivery, emphasizing novel delivery systems. Hopefully, this review would inspire scientists and researchers in the field of drug delivery across BBB to uncover new techniques and strategies to optimize drug delivery to the brain. Considering the anatomy, physiology, and pathophysiological functioning of the BBB in health and disease conditions, this review is focused on the controversies drawn from conclusions of recently published studies on issues such as the penetrability of nanoparticles into the brain, and whether active targeted drug delivery into the brain could be achieved with the use of nanoparticles. We also extended the review to cover novel non-nanoparticle strategies such as using viral and peptide vectors and other non-invasive techniques to enhance brain uptake of drugs.

**Graphical abstract:**

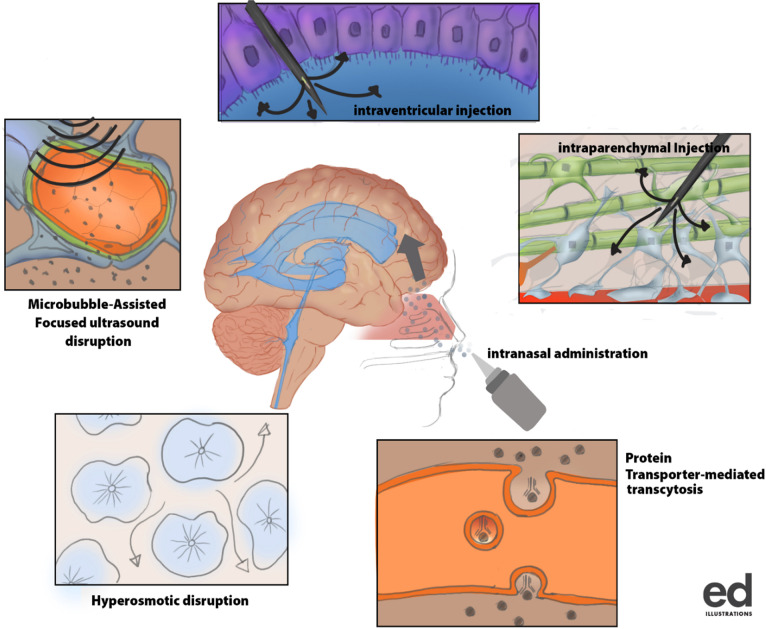

## INTRODUCTION

Brain diseases such as Alzheimer’s disease (AD), Parkinson’s disease (PD), stroke, and mental disorders are some of the most devastating and poorly treated diseases in the USA (1). These diseases are reported to be responsible for a high disability-adjusted life years (DALYs) in the USA and are the second leading cause of deaths globally (1, 2). The development of new medications for brain diseases has to be increased significantly as over 1.5 billion people worldwide have been estimated to suffer from at least one or more of these diseases in the next 15 years (2). However, drug development for these diseases has the poorest success rate due to the complexity of the brain, unwanted effects, and impermeability of the BBB (3). In addition, the lack of proper technologies to significantly enhance delivery of drugs across the BBB has hindered drug development for various brain diseases (4). Most research aimed at developing new chemical entities (NCE) for managing brain diseases has shown the effectiveness of small and large molecules in treating different brain diseases (5–9). However, only lipid-soluble small molecules (with small molecular weights < 400 Da) can successfully cross the BBB, while most large molecules cannot penetrate the brain endothelium. Even so, most of the small molecules that do penetrate the BBB are pumped out by multidrug resistant proteins. This has made the entry of drugs into the brain a huge challenge as only 5% of molecules for drug development are successful (10). With these in mind, understanding the modulation of the BBB permeability would fuel pharmaceutical interest in optimizing drug delivery to the brain.

The BBB is a naturally existing physiological barrier that prevents xenobiotics molecules into the brain parenchyma, and overcoming it has been a major challenge in the field of drug delivery or pharmaceutical industry (11). It has a unique selective permeability made up of a structurally distinct endothelial cell layer separating the brain from the blood with surrounding glial cells (11). Endothelial cells connect to the adjacent one by tight junctions serving as the paracellular route for the passage of polar molecules between neurons (12).

Several approaches in developing novel drug delivery techniques have exploited the organizational and functional characteristics of the BBB in health and some diseased conditions such as the use of niosomes for central nervous system (CNS) drug delivery (13). Several studies have demonstrated that brain diseases such as Alzheimer’s disease, brain cancer, and stroke may compromise BBB integrity resulting in a breakdown that exposes the brain parenchyma into leaks of neurotoxic components from blood. This activity leads to neuronal necrosis and degeneration of the axons (14, 15). Based on several mechanisms that undermine the BBB during injury, techniques have been derived to optimize CNS drug delivery using these “weaknesses” to reduce further secondary damage. Typical example is the treatment of brain tumor where the drug has to overcome two main existing barriers: the BBB and the recently documented blood–brain-tumor barrier (BBTB). This increases the challenge of successful delivery of drugs to the brain cancer (16).

Advances in biomedical sciences and technological developments have led to a better understanding of the pathophysiological mechanisms of neurological disorders. As such, there has been a tremendous advancement in genomics driving towards precision medicine, which will need effective delivery systems to optimize patient outcomes. In this domain, active targeting with nanotechnology, vectors, and brain permeability enhancers seem to be a breakthrough in effective brain drug delivery (17).

Recent data show that only one in ten global prescribed drugs covers CNS diseases, most of which are used in pain management and mental health-related diseases (Tables I and II) (18). The given data suggest that the CNS market is underperforming, with a major challenge being the inability to overcome the BBB. Thus, research needs to keep improving the development of novel formulations for drug delivery into the CNS. This review focuses on new strategies that have been investigated to deliver genes, therapeutic and diagnostic agents, to the brain across the BBB.

**Table I:** Novel CNS Drugs in Clinical Trials ( Source: Patel & Patel, 2017)

s/n	Drug candidate	Mechanism	Indication	Findings
1	Sorafenib + everolimus	Tyrosine kinase inhibitor	Brain tumor Glioblastoma Anaplastic glioma	Maximum dose tolerated to be 200 mg twice daily
2	Sunitinib (high-dose, intermittent)	Tyrosine (multiple) kinase inhibitor	Recurrent GBM	Promising outcomes if intermittent dosage of 300 mg compared to 5 0 mg of previous clinical trial is well tolerated
3	GnbAC1	Humanized IgG4 mAb targeting retroviral envelope	Relapsing remitting multiple sclerosis	No clear immunoregulatory effect in MS but showed remyelinating potential. Study showed safety and good tolerance of drug
4	Vandetanib + temozolomide; vandetanib + carboplatin	EGFR and VEGF receptor 2 inhibitor	GBM	Results unclear; well tolerated but efficacy not ascertained, study terminated
5	ABT-436	Vasopressin 1b receptor antagonist	Alcohol dependence	Greater percent days of abstinence than placebo group
6		Peptide receptor antagonist	Acute migraine	Pain freedom within 2 h of 50 and 25 mg; further research needed to determine long-term safety
7	SAGE-217	Positive allosteric modulator of GABA type A receptor	Major depressive disorder	Administered for 14 days resulted in reduction in depressive symptoms for 15 days but with more adverse events
8	Rimegepant	Calcitonin gene-related peptide receptor antagonist	Migraine	High percentage of patients free from pain and most bothersome symptoms
9	Anlotinib With STUPP Regimen	Inhibit both tumor angiogenesis and proliferation	Newly diagnosed and recurrent glioblastoma	
10	Aducanumab (BIIB037)	Human IgG1 monoclonal antibody against a conformational epitope found on Aβ	Alzheimer’s disease	Potentially beneficial being an immune checkpoint inhibitor
11	Selumetinib	Blocks proteins that allow tumor cells grow without stopping	Astrocytoma low-grade glioma	
12	Selumetinib	Blocks proteins that allow tumor cells grow without stopping	Neurofibromatosis type 1 and symptomatic inoperable plexiform neurofibromas	
13	Inotuzumab ozogamicin	Monoclonal antibody, linked to anti-cancer calicheamicin	Acute lymphoblastic leukemia CNS leukemia	Improvements in PN-related pain and motor impairment, durable tumor shrinkage

*GBM* glioblastoma multiforme; *AD* Alzheimer’s disease; *EGFR* epithelia growth factor receptor; *VEGF* vascular endothelial growth factor, *mAb* monoclonal antibody

**Table II:** Novel Approved CNS Drugs ( Source: Patel & Patel, 2017)

S/N	Brand name	Drug candidate	Mechanism	Indication	Type of formulation
1	Zembrace	Sumatriptan 5-HT	5-HT_1D_ and 5-HT_1B_ agonist	Migraine	Injection in disposable pen
2	Adzenys XR-ODT	Amphetamine	CNS stimulant	ADHD	Orally disintegrating tablet
3	Briviact	Brivaracetam	Bins to synaptic vesicles glycoprotein 2A	Epilepsy	Tablet, oral solution, injection
4	Rytary	Carbidopa/levodopa	DOPA decarboxylase inhibitor and DOPA	Parkinson’s disease	Extended-release capsules
5	Duopa	Carbidopa/levodopa	DOPA decarboxylase inhibitor and DOPA	Parkinson’s disease	Enteral suspension
6	Aptensio XR	Methylphenidate	CNS stimulant	ADHD	Extended-release capsules
7	Zulresso	Brexanolone	Positive allosteric modulation of GABA_A_ receptors	Postpartum Depression	IV infusion
8	Sunosi	Solriamfetol	Dopamine and norepinephrine reuptake inhibitor	Treat somnolence or obstructive sleep apnea	Oral tablet
9	Wakix	Pitolisant	Antagonist/inverse agonist at histamine-3 (H3) receptors	To treat excessive daytime sleepiness (EDS) in adult patients with narcolepsy	Oral Tablet
10	Nourianz	Istradefylline	Adenosine A_2A_ receptor antagonist	To treat adult patients with Parkinson’s disease experiencing “off” episodes	Film Coated tablet
11	Reyvow	Lasmiditan	Serotonin 5-HT _1F_ receptor agonist	For the acute treatment of migraine with or without aura, in adults	Tablet
12	Xcopri	Cenobamate	Positive allosteric modulator of GABA_A_ ion channel	To treat partial onset seizures	Tablet
13	Caplyta	Lumateperone tosylate	Antagonist activity at the central serotonin 5-HT_2A_ and postsynaptic dopamine D_2_ receptors	To treat schizophrenia	Capsule
14	Dayvigo	Lemborexant	Orexin A and B receptors antagonist	Treat insomnia	Tablet
15	Ubrelvy	Ubrogepant	Calcitonin gene–related peptide receptor antagonist	To treat acute treatment of migraine with or without aura in adults	Tablet

*AMPA* α-amino-3-hydroxy-5-methyl-4-isoxazolepropionic acid, *ADHD* attention deficit hyperactive disorder, *DOPA* L-3,4-dihydroxyphenylalanine, *CNS* central nervous system

## ANATOMY AND PHYSIOLOGY OF THE BBB

The BBB is made up of endothelial cells of the CNS sealed together by tight junctions forming a physical barrier that prevents the passage of toxic substances into the brain (19). The complex ramified arterial network of the circle of Willis (blood capillary network supplying the brain) made from the merging of the internal carotid and vertebral arteries provides a rich blood supply to the brain parenchyma (20, 21). The endothelial cells are specialized in maintaining communication and exchange of materials between CNS neurons and neuroglia cells while ensuring a continuous, non-fenestrated basal lamina (22).

**Fig. 1:**
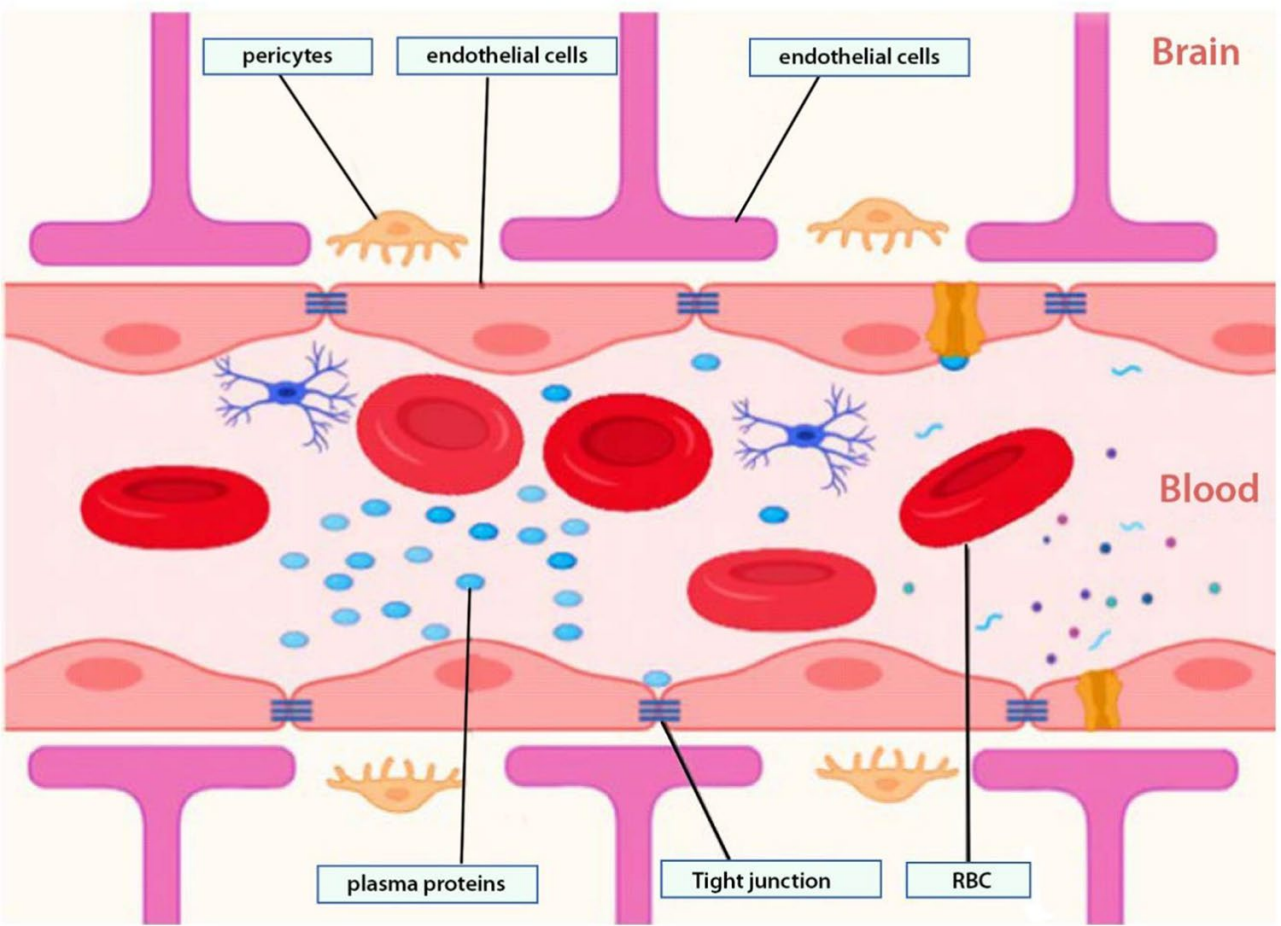
Schematic view of the BBB. Microscopic view of the organization of the blood–brain barrier from the luminal surface (with blood and tissue fluids including drugs) and the abluminal surface, which is made up of the parenchyma of the brain (neurons and neuroglia). The barrier is made up of endothelial cell layers (connected by tight junctions), glia cells, and pericytes

The development and maturation of the BBB are ensured by astrocytes and pericytes which secrete sonic hedgehog and retinoic signaling proteins responsible for maintaining its integrity (21, 23). With the growth of the BBB, the perivascular space is covered by foot-like processes projecting from the astrocytes. At the same time, the pericytes provide the scaffold of the structural integrity of the basal lamina (Fig. 1) (24). Some areas (circumventricular regions) such as the area postrema are deficient of the BBB and thus allow plasma proteins and infections to access the brain.

## TRANSPORTERS EXPRESSED AT THE BBB

**Fig. 2:**
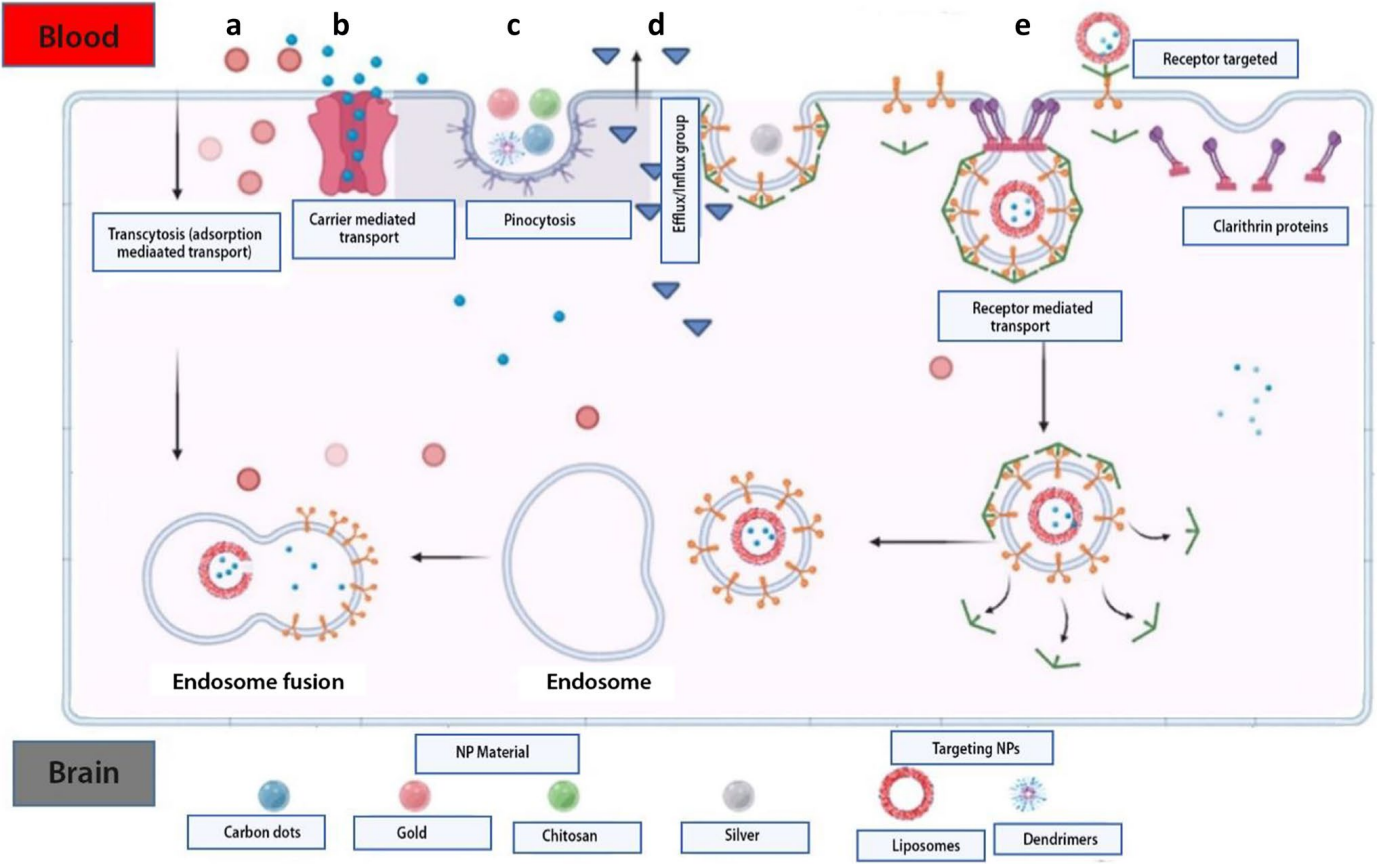
Methods of transporting materials across the BBB. Naturally occurring material used as NP via cellular adsorption–mediated transport by using surface charges (**a**). Delivery of small molecules using carrier-mediated transport through membrane proteins (**b**). Endocytosis transport of natural inorganic nanoparticles in the cell (**c**). Efflux pump mechanism posing drug resistance into the brain (**d**). Nanoparticle delivery using surface receptors such as transferrin and LDL targeting receptors (**e**)

The BBB has two main functions of protecting (or shielding) the brain and transportation of substances across. It may allow substances to cross into the brain by one of the following ways: (1) paracellular transport (which involves passage between endothelial cells) and (2) transcellular transport (involving passage within or through the cell from the luminal to the abluminal surface of the endothelial cell into the parenchyma of the brain). Tight junctions located between endothelial cells prevent the passage of molecules through the paracellular route, while the transcellular route allows for the passage of molecules based on their electrochemical gradient (concentration, electrical charge, and lipophilicity). Active transport uses adenosine triphosphate (ATP) molecules as a source of energy to drive molecules against the concentration gradient across the BBB. These molecules are vastly less lipophilic and ferried across the BBB by processes such as (A) pericyte transporters; (B) endothelial ion transport (sodium pump, potassium channels, and calcium transporters); (C) endothelial solute carrier-mediated transport (carbohydrate, amino acid, monocarboxylate, hormonal, fatty acid, nucleotide, and organic anion and cation transporters); (D) endothelial active efflux (ATP-binding cassettes), and (E) endothelial receptor–mediated transport (transferrin, insulin, and lipoprotein transporters) (14) (Fig. 2).

### Factors Influencing Passage of Drug Molecules Across BBB

The BBB is a heterogeneous barrier with different expression levels of active efflux and influx transporters making drug penetration dependent on expression levels (10, 25). Importantly, the ability of drugs to cross the BBB is based on several factors ranging from the drug’s physicochemical properties, protein binding, cerebral blood flow, drug elimination, and integrity of the barrier.

First, physicochemical properties such as lipophilicity, hydrogen bonding, particle size, and surface charge influence the permeability of drug molecules across the BBB. For a drug to successfully reach its target in the brain, it must be capable of partitioning itself between lipophilicity and hydrophilicity phases. Highly lipophilic drugs risk being sequestered within or between the cell membrane, reducing their ability to reach the brain parenchyma. Likewise, very hydrophilic drugs cannot pass through the high lipid-soluble cell membrane. This makes the partition coefficient of a drug an important parameter for brain drug delivery (10).

Second, the relationship between BBB permeability and drug particle size can be predicted with the greatest accuracy for small molecules as well as small biologics like peptides. Peptides exist in simple and complex forms. Their permeability across the BBB is highly influenced by secondary structure folding in a manner that “hides” charges present on their primary structures leading to increase lipophilicity.

Lastly, drug molecules can be categorized into flow-dependent and flow-independent based on their extractability from circulation (26). High flow-dependent drugs will rely solely on cerebral blood flow for proper brain delivery despite its physicochemical properties; thus, increased blood flow increases the amount of drug reaching the BBB.

## CHALLENGES FOR DRUG TRANSPORT ACROSS THE BBB

The protective role of the BBB serves to prevent the movement of macromolecules, pathogens, and neurotoxins from getting into the brain (20). Notwithstanding, it also significantly inhibits the passage of therapeutic moieties from reaching their targets in the brain. As much as 90% of small-molecule drugs and larger therapeutic drugs are prevented from crossing the BBB (20). First, the presence of efflux proteins such as P-glycoprotein, multidrug resistance protein-1 (MRP-1), and ATP-binding cassette (ABC) transport proteins may actively exclude out the drug from the brain (27). These active efflux transporters can recognize and pump out as much as 60% of all marketed drugs contributing to drug resistance (28). Second, metabolic degradation has significantly reduced the accumulation of drugs in the brain, therefore reducing drug efficiency (20, 29).

Third, the presence of specific proteins such as tight junctions (TJs) and adherens junctions (AJs) is responsible for the strong cohesiveness of endothelial cells throughout the barrier. As endothelial cells are encompassed by a basal lamina, collagen, and heparin sulfate, they can be intriguing targets for drug transport. This barrier is responsible for its very low permeability, limiting the drugs from entering and obstructing the therapeutic effects in the CNS.

Several strategies have been employed in the past and recent years to improve the delivery of drugs across the BBB into the CNS. These range from disrupting the BBB physically and chemically, bypassing the BBB, and inhibiting efflux proteins, all to improve delivery.

## CURRENT APPROACHES FOR INCREASING BRAIN PENETRATION OF DRUGS

**Fig. 3:**
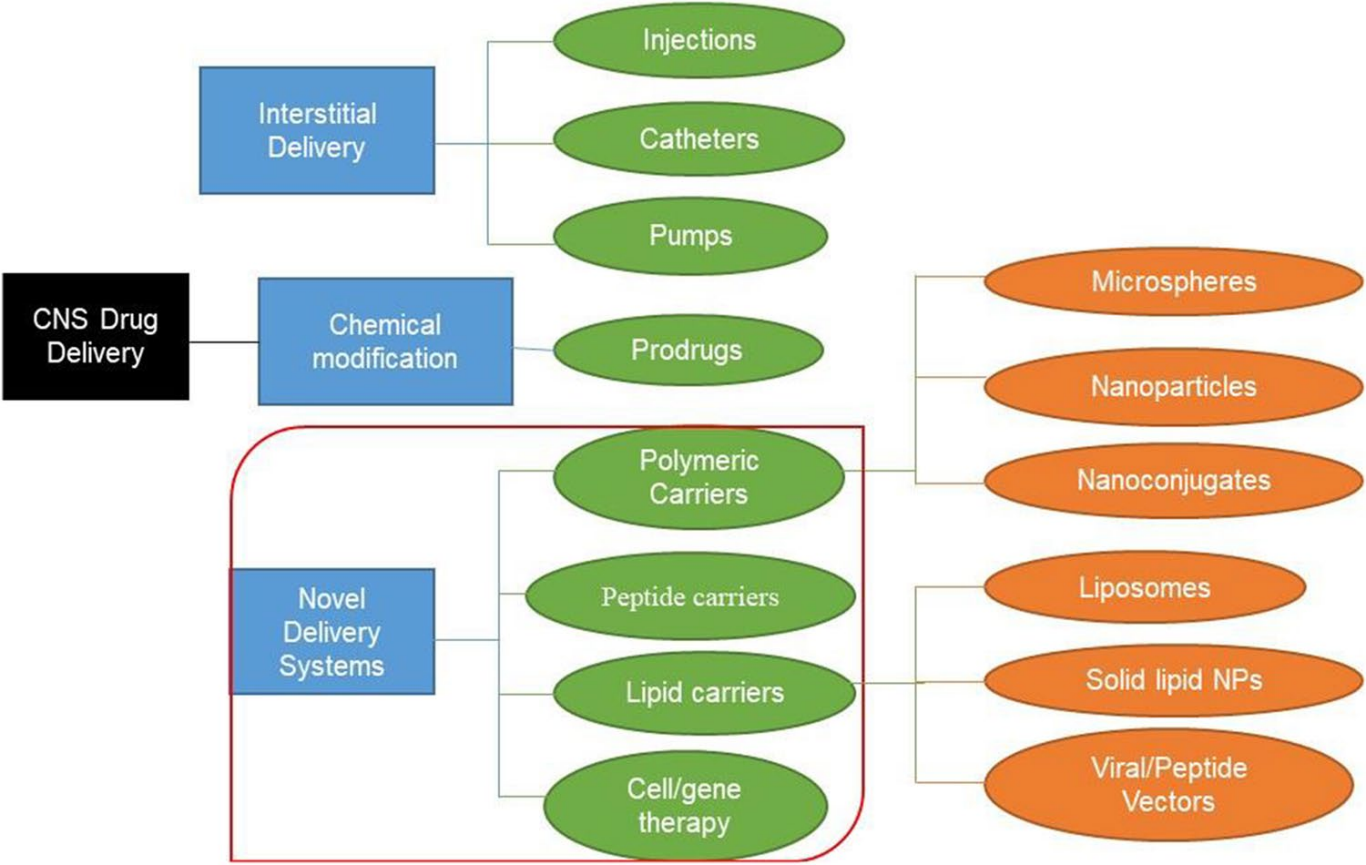
Brain drug delivery algorithm. It is mainly divided into three components made of interstitial, chemical modification, and novel delivery methods. Each of these components is subdivided into different categories (green color) and methods (orange color). The novel delivery component consists of 4 categories which include polymeric, peptide, and lipid carriers as well as gene therapy technique. Meanwhile, the polymeric carrier system involves the use of nanospheres, nanoparticles, and nanoconjugates, the lipid carrier system consists of liposomes, solid lipids, and viral/peptide vectors delivery systems

The knowledge and understanding of different properties and types of molecules are key to diagnosing and managing several CNS diseases. Researchers are currently harnessing the power of drug delivery systems to study the regulation of the cellular microenvironment and improve the efficiency of drug delivery to the brain (30). Recently, nanocarriers have been used in combination with other delivery approaches to improve CNS drug delivery to treat diseases such as neurodegeneration and brain cancer (31) (Fig. 3 and Table III).

**Table III:** Novel CNS Nanoparticle Delivery Systems

S/N	Delivery system	Drug/procedure	Status	Mechanism
1	Viral vector	Preprodynorphin gene	Preclinical	AAV-mediated expression of preprodynorphin in the epileptogenic hippocampus
2	SLN	Coumarin	Preclinical	Borneol-modified solid lipid nanoparticle loaded with coumarin
3	Polymeric NPs	Elvitegravir	Preclinical	PLGA-EVG NP inhibits efflux proteins to optimize CNS delivery
4	Intranasal	RVG29	Preclinical	Targeted PLGA Nanoparticles loaded with rabies virus glycoprotein (RVG29)
5	Targeted NPs	Nevirapine	Preclinical	Polycaprolactone NPs bind to LDL receptors to optimize delivery by RMT
6	Targeted SLN	Zidovudine	Clinical	AZT-SLN receptor specific RMT across the BBB
7	Non-invasive	MRgFUS	Clinical	Non-invasive BBB disruption at the primary motor cortex using MR-guided Focused Ultrasound
8	Vector	AdV-tk	Phase I	Gene-mediated cytotoxic immunotherapy through local delivery of AdV-tk

*SLN* solid lipid nanoparticle, *MRgFUS* magnetic resonance–guided focused ultrasound, *AdV−tk* adenovirus−mediated thymidine kinase

### Targeting Centers and Receptors

A more robust way to circumvent the BBB is to allow drugs to be taken up past endothelial cells by receptor-mediated endocytosis (RME) and is the primary method by which most novel drug delivery systems exploit to gain access to the brain. In RME, targeting can be achieved by directly coupling the targeting ligand to a drug molecule or a delivery carrier system. The targeting ligand binds to a receptor found on the surface of the vascular endothelial cells of the BBB to shuttle the drug or molecule across. Some widely used targets include transferrin, insulin, low-density lipoprotein, and diphtheria toxin receptors (32–35). After endocytosis, transcytosis takes place to transport the drug molecules of the carrier system to the abluminal surface of the brain parenchyma (Fig. 2).

#### Receptor-Mediated Transport (RMT)

RMT has been demonstrated to utilize several receptors such as low-density lipoprotein receptor (LDLR), transferrin receptor (TfR), insulin receptor (INSR), and insulin-like growth factor receptors (IGFIR and IGFRIIR), low-density lipoprotein receptor-related protein 1 (LRP1), receptor for advanced glycation end products (AGER), and leptin receptor (LEPR) as well as the receptor for BBB-crossing antibody FC5 (TMEM30A, isoforms 1 and 2) (5).

##### Transferrin Receptor (TfR) 

TfR is the most widely exploited in preclinical and clinical studies among all the receptors (36). Transferrin is an iron-binding blood plasma glycoprotein that regulates the levels of free iron in biological fluids. It binds tightly and reversibly to iron and remains the major source of iron delivery to the neurons. This receptor is highly expressed on endothelial cells of the BBB, immature red blood cells, placenta tissue, hepatocytes, and most malignant cells of CNS origin (37, 38). Despite the unsuccessful clinical constructs of targeting transferrin receptors (TfR), it has remained a popular target. However, cargo-loaded liposomes and gold nanoparticles functionalized with antibody ligands for TfR have shown promising results in the delivery of NPs into the CNS (39, 40). This seems to be a more advanced method of improving transferrin receptors by binding to highly expressed in the endothelial cells of the BBB, through interaction with ligands functionalized on NPs for clinical trials.

##### Low-Density Lipoprotein Receptor-Related Protein 1 (LRP1)

LRP1 is a crucial receptor widely expressed in many tissues with diverse roles in different biological processes (6). It is also an essential receptor involved in amyloid beta-protein (Aβ) clearance in cerebral cells, across the BBB and in peripheral Aβ-degrading organs (6). In recent studies, researchers have been able to simulate *in vitro* BBB using amniotic fluid–derived induced pluripotent stem cells (AF-iPSC) while demonstrating their superiority in transcytosis using acetylated low-density lipoprotein-like receptors (LRP1) (5). These LRP1 and high-density lipoproteins make up a complex molecule known as apolipoprotein E (ApoE), which transports cholesterol and other lipids across the BBB (41, 42). To take advantage of this, grafting nanoparticles or drugs with ApoE could target LRP1 in the brain. These receptors could be triggered with antibodies to optimize CNS drug delivery through receptor-mediated transcytosis in an otherwise typical BBB (5, 43, 44). The ApoE receptors have been identified as acetylated low-density lipoproteins (LDL) which binds to antibodies FC5-Fc and IGF1R5-Fc allowing for endocytosis across the BBB (5). Although the above study did not mention the presence of tight junctions, Li and colleagues performed a similar study with a model that included tight junctions making it complete and reproducible for further studies to screen for drugs that could penetrate the BBB for CNS drug delivery (45). These *in vitro* studies have been demonstrated with preclinical mice studies involving the conjugation of biodegradable polymers (polyethylene glycol-polylactic-co-glycolic acid) doxorubicin-loaded micelles targeted interleukin-6 receptor (I6P8 receptor) to optimize CNS delivery for treatment of glioma (46). It has also been demonstrated that LRP1 is bound to specific ligands, for instance, angiopep-2, a member of a family of peptides derived from the Kunitz domains of aprotinin and other human proteins. They aid in the transport of drugs across the BBB from blood to brain (47).

Notwithstanding, the TfR and ApoE receptor targeting are not without shortcomings (like high endogenous levels of transferrin *in vivo* saturating the TfR receptors and bulky nature of the ApoE receptor, respectively); however, much of these limitations have been reduced with the use of antibodies against the TfR receptor (example OX26). OX26 is a monoclonal antibody that binds selectively to a specific epitope of the rat TfR different from the transferrin-binding site (46).

#### Carrier-Mediated Transport (CMT)

CMT exploits the route used by endogenous molecules to cross the BBB. These carriers could be identified as those that aid in the passage of molecules such as glucose, amino acids, and vitamins into the CNS. This method of drug delivery has been widely reported in *in vitro* studies but limited data in preclinical studies (7, 48, 49). Nanoparticles and liposomal formulations are two widely used for CNS drug delivery, performing their role through CMT. These are small vesicle-like molecules comprising one or more concentric bilayers of phospholipids separated by aqueous compartments (50). Also, biomolecules such as glucose conjugated with thiamine disulfide system and the drug venlafaxine showed increased clinical efficiency more than five times as compared to the parent drug alone or the drug-glucose conjugate in treating major depression (51). The GLUT1 receptor being a bidirectional transport system permits glucose to move either way. To stop glucose-venlafaxine conjugate from diffusing back to the blood, the thiamine disulfide system was introduced to modify the conjugate and lock in the drug within the CNS. The conjugation of Ibuprofen with glucose and vitamin C for improved CNS delivery of Ibuprofen for the neurodegenerative disease was shown to be highly effective with increased anti-inflammatory effects of Ibuprofen recorded compared to when the drug was used alone (52).

### Vectors

This method involves using genetically modified plasmids in viruses that cross the BBB in delivering siRNA and shRNA plasmids into CNS for gene therapy. The main challenge is the permeability of the viral vectors across BBB for brain drug delivery without adulterating the biologics. There are two main vectors currently in research: the viral and peptide vectors.

#### Viral Vectors

Little has been done/understood in the use of viruses as carriers or vehicles for drug delivery across the BBB. However, several molecules have been identified on the luminal surface of the BBB, which can interact with some ligands present on some viruses such as the adeno-associated virus (AAV) (8). The intrinsic properties of these pathogens to breach the integrity of the BBB have been exploited by researchers to improve delivery. Ryan and colleagues demonstrated the use of immunotherapeutic to clear amyloid-beta (Aß) deposited in the brain of transgenic mice to enhance cognitive function in Alzheimer’s disease–induced mice (53). In this study, recombinant adeno-associated virus (rAAV) was cloned from an isolate of human single-chain variable fragment (scFV) antibody library (a new antibody specific for Aß). Transgenic mice induced with Aß deposits, neurofibrillary tangles, synapse, and neuronal loss were infused with the rAAV vector encoding Aß-scFV into the hippocampus of the brain. Mice receiving rAAV-AB-scFV had significantly lower levels of Aß and improved cognitive function as measured by the Morris Water maze (an apparatus designed for behavioral studies in rodents) than controls (54).

Another study by Alonso and colleagues, using chimeric adeno-associated virus (AAV) containing a coding region for LacZ genes, demonstrated specific, localized, and efficient expression of intravenously administered transgenes by ultrasound-assisted BBB disruption. The expression of the LacZ gene was confirmed by histochemical staining showing cells with enzymatically active proteins and double immunofluorescence with antibodies against bacteria LacZ in neurons (55). Dos Santos and colleagues, in a preclinical trial, demonstrated that without any detectable cellular damage, plasmids with beta-galactosidase were transfected into mice brains. This event leads to the production and modulation of beta-galactosidase in the brain to treat neurodegenerative disease linked with lysosomal storage disorder (56). This study employed dual-functional liposomes for efficient neuronal transfection in combination with transferrin receptors to enhance CNS penetration. *In vitro* studies have confirmed the ability of dual-functional liposomes to cross the BBB and transfect primary neuronal cells. Also, *in vivo* quantification of intravenously administered dual-functional liposomes loaded with a transgene (ß-galactosidase) demonstrated expressive accumulation in mice brains without toxicity (56).

**Fig. 4:**
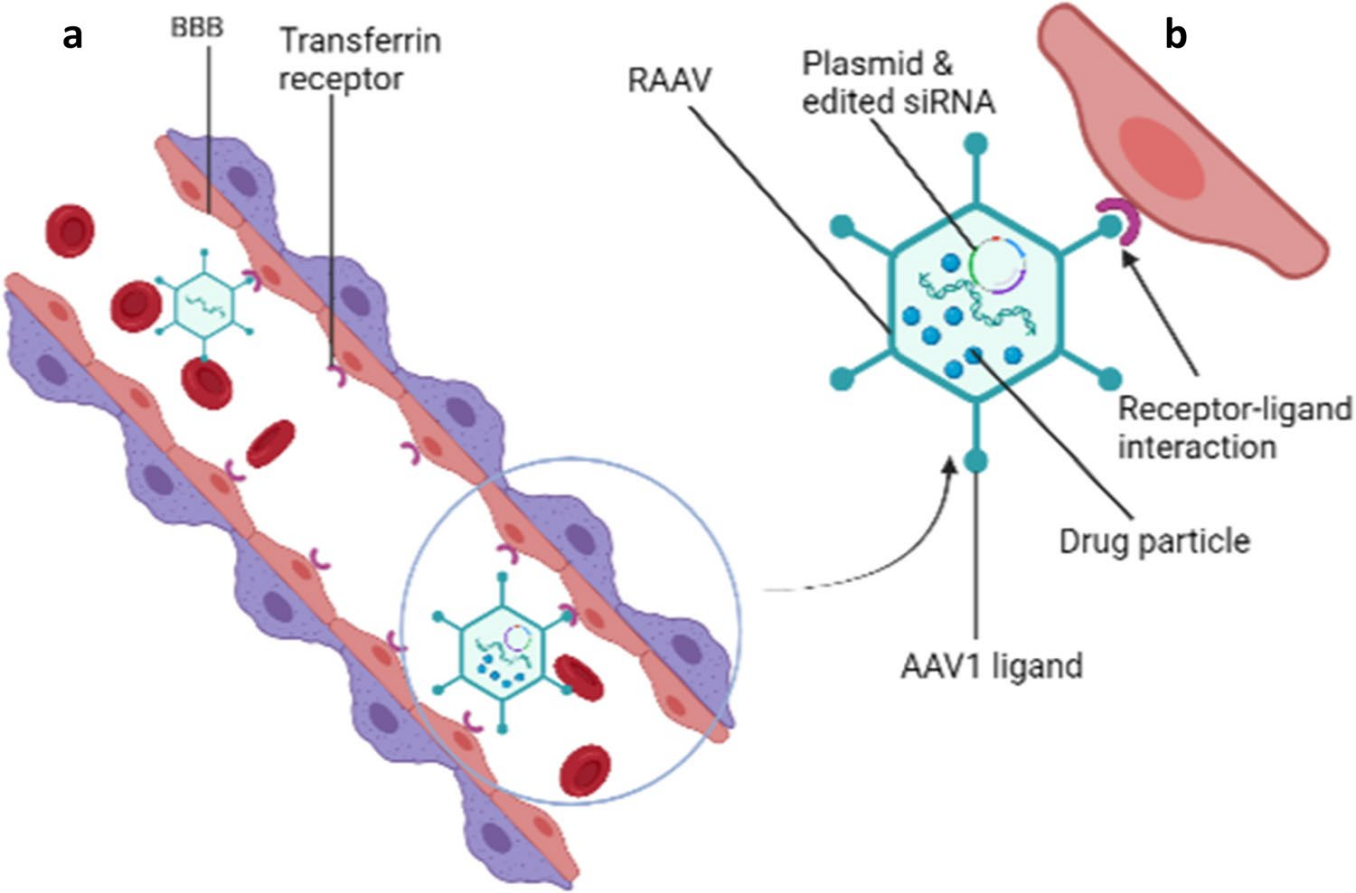
Recombinant adeno-associated virus (raav) as a vector for drug delivery in the brain (**a**). Delivery of raav drug-loaded particles to the brain by receptor-mediated transcytosis (**b**)

**Fig. 5:**
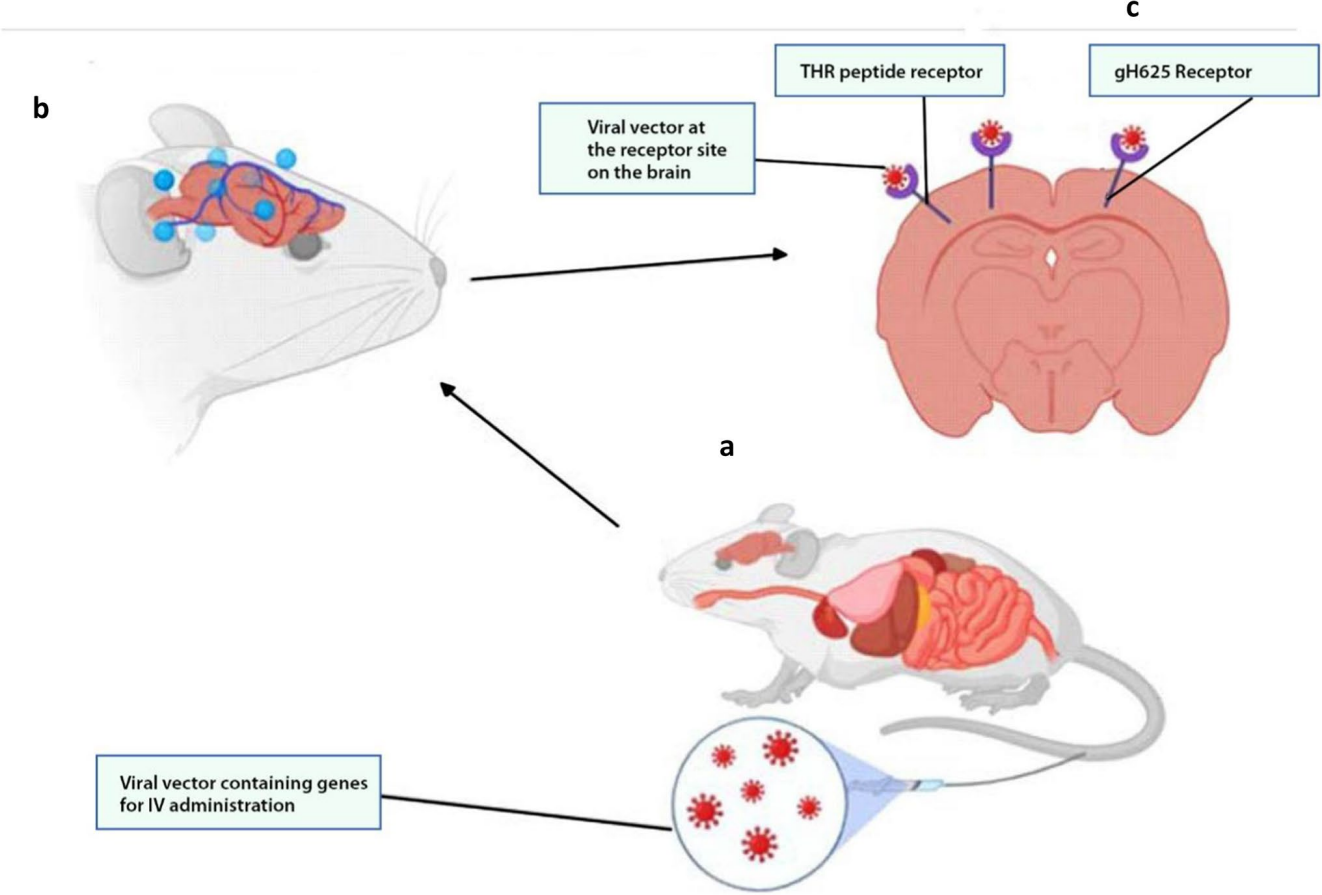
Nanoparticle delivery system for drug delivery into the CNS. *In vitro* and *in vivo* drug-loaded nanoparticle delivery system into the CNS (**a**) and (**b**), respectively. Passive diffusion of the drug to the brain parenchyma (**c**)

Again, viral vectors have been applied in preclinical and clinical trials for gene therapy for CNS diseases. Studies conducted by Zhang and colleagues identified several BBB shuttle peptides (THR) that significantly enhanced the transduction of AAV8 after systemic administration (8). The shuttle peptide expression enhanced direct binding to the endothelial cells and transduction of AAV8 across the BBB in a dose-dependent manner to target neurons in the CNS (Figs. 4 and 5) (8).

Another study focused on comparative analysis of different capsid domains on 36 chimeric capsid sequences using AAV serotypes that neither crossed the BBB (AAVrh.10) nor vasculature (AAV1) was conducted by Albright and colleagues (57). The study showed that a cluster of residues sorted on the AAVrh.10 capsid enhanced transport across brain vasculature and neuronal transduction in mice (57). Though not well described, the mechanism used receptor-mediated transcytosis to transfect capsid-protected genetic particles into the brain. This shows a road map for the possible exploitation of AAV capsid for CNS gene transfer.

More studies have been carried out recently on the transduction of genes across the BBB using different AAV serotypes with AAV9 being the latest (Fig. 4) (8, 57, 58). Meanwhile, *in vitro* and animal studies have shown the AAV serotypes to effectively transduce genetic information across the BBB with a poorly described mechanism. Alimonti and colleagues showed a putative Zika virus cellular entry receptor on the BBB *in vitro*. They used human-induced pluripotent stem cell–derived BBB models to demonstrate how the Zika virus infects the brain endothelial cells without compromising the integrity of the barrier (9). Based on the findings, the Zika virus could be used as a potential viral vector after editing the pathogenic genes and transfecting them with gene therapies for CNS delivery. It has been scientifically demonstrated that the gH625 proteins obtained from the Herpes Simplex virus 1 have improved the delivery of liposomal-loaded drugs into the CNS (Fig. 5).

A recent breakthrough for effective delivery of drugs across BBB for treating CNS diseases was using a carrier molecule composed of modified peptides from a bacteriophage Fd. It has been shown to target the brain by binding to transferrin receptors followed by crossing BBB and finally reaching neurons and microglia cells in the brain (59). This process allows for minimally invasive combination delivery through intravenous injection of various drugs, peptides, and nucleic acid therapeutics to the brain.

Also, retrograde gene transfer using lentivirus vector has been documented to be efficient in drug delivery into primate brain (60). Foreign genes (green fluorescent protein (GFP)) were introduced into the animal’s brain using pseudotyped lentiviral vector (Fug-E), and later immunofluorescence analysis found many GFP immune reactive neurons in the striatum of the primate. This shows that lentivirus is an excellent viral vector for gene therapy in the CNS, and this could be a breakthrough in the clinical management of aggressive brain tumor with highly expressed P-glycoproteins.

More importantly, recent procedures have identified and used appropriate receptors to which viral particle ligands can interact at the BBB and enable receptor-mediated transfection. This has thus eased the delivery of the plasmid through the intravenous route instead of other invasive methods. Many gene therapies have gone through clinical trial phase I; however; just a few have made it through phase III due to concerns on transduction efficiency, specificity of the target, safety profile, and level of transgene expression (61). More techniques employed recently have addressed the aforementioned issues to improve clinical outcomes. Phase I dose-escalation studies have been performed by gene-mediated cytotoxic immunotherapy through local delivery of aglatimagene besadenovec (AdV-tk) in pediatric gliomas with promising outcome (62).

#### Peptide Vectors

Many research approaches have used peptides as a molecular Trojan horse to target the BBB for CNS drug delivery. Recombinant fusion proteins, naturally occurring proteins (melanotransferrin, aprotinin, angiopeptide, and apolipoprotein B) are some of the proteins that have been used as ligands, receptor targets, and vectors for the delivery of recombinant enzymes into the CNS (63, 64). Annika and colleagues performed an *in vitro* comparative study of five peptide vectors. In this study, five different brain-targeting peptides were used to promote the brain delivery of the lysosomal enzyme arylsulfatase A (ASA). The transduction domain of HIV TAT protein, angiopeptide (Ang-2), and receptor-binding domain of human apolipoprotein B and E (two versions: ApoE-I and ApoE-II) were generated and chimerically linked unto ASA. Transendothelial transfer of the enzyme into the CNS was optimal when transported by ApoE-II compared to other fusion proteins through endocytosis by mannose-6-phosphate receptor (63). This study confirms the possibility of using different plasma proteins capable of penetrating the BBB or circumventing the CNS as possible routes of improving brain delivery.

Currently, peptide vectors such as angiopep-2, a 19-amino acid derivative of aprotinin, are in the clinical trial phase as an angiopep-2-drug conjugate to target the BBB for improved drug delivery to the brain. Angiopep-2 can trigger transcytosis and traverse the BBB by targeting low-density lipoprotein-related protein-1 (LRP-1) receptors expressed on the brain capillary endothelial cells (38). In another study, surfactant-coated biodegradable nanoparticles were used to increase the delivery of ASA in the brain. This approach showed a significant increase in BBB penetration through high-affinity binding via the streptavidin–biotin system (65).

We cannot overemphasize that vectors for gene therapy and delivery into CNS are valuable avenues to be exploited for a potential breakthrough in medicine because of their high transduction efficiency penetrating cells in the CNS and do not require cell division. Further, some vectors such as AAV can integrate into the host genome non-pathogenic genes and non-inflammatory cascade stimulation (66). While vectors play a significant role in delivering drugs to the brain, some associated shortcomings such as poor integration, immunogenicity, poor replication competence, and poor/missed targeting are some of the challenges. In addition, the small sizes of these vehicles make loading a challenge especially as large molecules cannot be loaded with success.

### Nanoparticle Delivery Systems

Nanoparticles (NPs) are small dimension (of a few hundred nanometers) size materials that can interact with biological entities in a fundamentally different way from non-structural materials. These techniques have been employed over the years wherein different substances are combined using different methods to entrap drugs and maximize drug delivery. NPs technology has also been used to improve drug delivery into the CNS enabling delivery without physically disrupting the BBB. Different forms have been made to improve binding and passage across the BBB, like polymeric NPs, liposomal NPs, and inorganic systems lately. The main drawback with its use is the constant activity of P-gp on the BBB in pumping out drug-loaded NPs which have been able to pass the BBB (67).

**Fig. 6:**
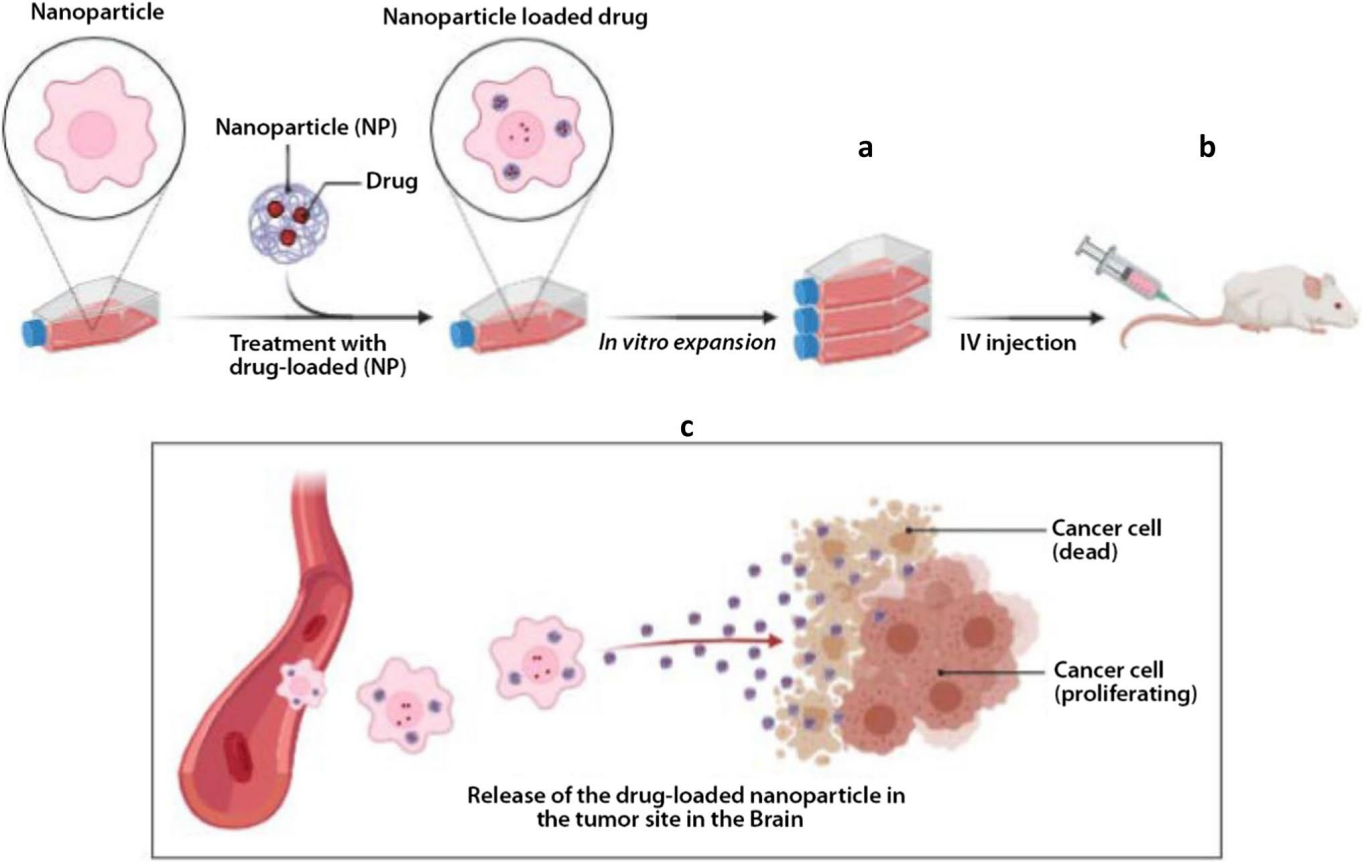
Methods of viral vector delivery of gene therapy into the CNS. *In vitro* delivery of drug-loaded nanoparticles (**a**). Intravenous (IV) administration of drug-loaded nanoparticles in mice (**b**). Release of the drug-loaded nanoparticle into the brain (**c**)

Recently, various designs of the NPs have been made to take advantage of the morphology and physiology of the BBB to improve drug-loaded NPs into the CNS. This often involves a combination of approaches to optimize drug delivery (Fig. 6).

#### Liposomal Nanoparticles

Liposomes are spherical molecules made up of single amphiphilic lipid bilayers loaded with drugs for delivery. They have been used over the years for research and are currently approved by FDA as a drug delivery system. Conventional unmodified liposomes consisting of naturally occurring phospholipids and cholesterol suffer from high systemic plasma clearance. They are generally cleared from the system circulation after administration by immune system macrophages of the reticuloendothelial system (68).

Numerous strategies have been adopted to overcome the short half-life of liposomes in the circulation such as grafting the liposomes with gangliosides or PEG. Conventional liposomes grafted with a PEG layer are considered to be sterically stable and safe from degradation. Grafting liposomes with different targeting peptides or antibodies for targeting strategies has been a successful drug and gene delivery tool. Huwyler and colleagues (69) presented the first preclinical trial to use liposomal drug delivery to target the brain. They used PEGylated liposomes conjugated to OX26 monoclonal antibody to enter the brain via transferrin receptor-mediated endocytosis successfully.

Similarly, peptide-grafted liposomes facilitated a six-fold increase in ferulic acid uptake into the brain. Another study demonstrated that transferrin and cell-penetrating peptide-grafted liposomes increase CNS accumulation by 10- and 2.7-fold high, respectively, for doxorubicin and erlotinib in mice studies (70). Also, liposomes have been shown to increasingly reduce glioma viability by 80% when loaded with genistein and administered intravenously as compared with the drug alone (71). Furthermore, liposomes have been employed in a recent study to transport and deliver sono-active chlorine and autophagy inhibitor (hydroxychloroquine) for the transient and reversible disruption of the BBB to improve uptake of chemotherapeutic drugs for the treatment of gliomas in mice. This indicated apoptosis and MAPK/p38-PINK1-PRKN dependent mitophagy through which the antioxidant could relieve the sonotoxic effects of the drug by activating the MAPK/p38 (72).

Liposomes are functionalized with viral proteins such as gH625 obtained from the herpes simplex virus 1 to promote the uptake of a neuroprotective pituitary adenylate cyclase-activating polypeptide (PACAP) into the brain. In this study, the liposomes were demonstrated to be non-toxic to the endothelial cells but rather the presence of gH625 improved the efficiency of liposomes to deliver PACAP to the mouse brain (73). It is important to note that liposomes on their own are a good delivery system for CNS penetration but functionalizing the surface may significantly improve their performance.

#### Nanostructured Lipid Carriers (NLC)

NLCs came up as the second generation of lipid nanoparticles to overcome the limitations of first-generation such as solid lipid nanoparticles (SLNs). Biodegradable and compatible lipids (solid and liquid) and emulsifiers are used for the preparation of NLCs. Liquid lipids (oil) incorporation causes structural imperfections of solid lipids leading to a less ordered crystalline arrangement which avert drug leakage and furnish a high drug load (74).

NLCs have been broadly studied and used in reducing toxicity of chemotherapeutics. Currently, in the field of brain delivery, it has greatly increased the delivery of previously effluxed-resistant drugs into the brain (75).

It is currently one of the safest, stable, biocompatible, and cost-effective drug carrier systems with high encapsulation efficiency for drug delivery in the brain. The unique structural properties of NLC make them ideal carrier systems for controlled drug release with a wide range of targeting, high drug loading capacity with little toxicity, and non-irritant behaviour (76). NLC has been used to improve antiretroviral drug (indinavir) delivery to the brain delivery (77). This study was conducted by Karami and colleagues (77) where lactoferrin nanoemulsions were developed to deliver indinavir into the brain of rats infected with HIV. The outcome of this study showed remarkable brain delivery enhancement with more than 400 times CNS uptake and clearance (77). In addition, both lipophilic and bioactive compounds have been developed to increase the permeability of drugs across BBB. A study with tanshinone-1, a compound reported to having cytotoxicity to a variety of tumor cells (36), was formulated into tanshinone-1 nanoemulsion with surface functionalized ligands and demonstrated a significant increase in the uptake of coumarin-6 in the brain of rodents (78).

While drug delivery into the brain has primarily focused on intravenous (IV) administration, other studies focusing on nasal and oral administrations have been exploited and proven safe and efficient (79, 80). Methylthioadenosine-loaded NLC was used in the treatment of multiple sclerosis in cuprizone-induced demyelinated mice (81).

#### Polymeric Nanoparticles (NPs)

Polymeric NPs can be prepared from a plethora of monomers and using various polymerization techniques, and their properties can be tuned depending on their specific applications (82). The most common polymeric NP systems that have been exploited for brain targeting, are synthetic polymeric NPs, natural-based polymeric NPs, and hybrid NPs (82).

Polymeric NPs have attracted considerable interest over recent years due to their properties resulting from their small sizes. Advantages of polymeric NPs as drug carriers include their potential use for controlled release, the ability to protect drug and other molecules with biological activity against the environment, improve their bioavailability and therapeutic index (83, 84). With the rising incidence of CNS disorders worldwide, it is important to employ polymeric NP as a delivery system to improve drug permeability across the BBB (85, 86). While polycyanoacrylate NPs have been used in synergy with mannose for the delivery of donepezil (cognition-enhancing medication) to the brain for the treatment of Alzheimer’s disease (87), poly (lactide-co-glycolide) (PLGA) NPs, in a recent study, have been used to encapsulate doxorubicin for *in vitro* treatment of gliomas and delivery of siRNA across the BBB (85, 88). These polymeric NPs bind to receptors such as transferrin and OX26 to improve receptor-mediated transport across the BBB (85–88). In another study, dual targeting of astrocytes using antibody-grafted chitosan nanoparticle–loaded small interfering RNA (siRNA) demonstrated significant permeability across the BBB to inhibit HIV replication in the brain (89). The antibodies used were transferrin and bradykinin B2, specifically bound to TfR and bradykinin receptor (B2R), respectively, to deliver the siRNA to astrocytes as potential targeting ligands. The combined use of these antibodies for gene delivery revealed optimal cellular uptake and gene silencing efficiency in astrocytes. We view this as a promising research area with potential benefits in the treatment of CNS diseases (89).

#### Inorganic Nanostructured Molecules

Many inorganic molecules have been used to design nanoparticles for CNS drug delivery, and the most common ones used are gold, silver, and silica. Gold nanoparticles (AuNPs) have been used for delivery in various parts of the body (90). They have shown the potentiality to penetrate the BBB and interact with the CNS without toxicity by enhanced permeability and retention effect. When functionalized with polymers and ligands, their penetration via receptor-mediated delivery becomes highly optimal (90). Surface modification of AuNPs with brain-targeted exosomes has demonstrated good binding to the brain cells under a laminar flow of blood to enhance transport across the BBB. *In vivo* studies further showed accumulation of exosome-coated AuNPs in the mouse brain following intravenous injection and traced with bioluminescent imaging (91). This seems to be a more efficient method of brain targeting with inorganic molecules with minimal toxicity. In addition, silver is another inorganic molecule that has been the most profit-oriented precious metal used in preparing nanoparticle (AgNPs) delivery systems to bind unto serotonin for CNS drug delivery (92). AgNPs have been shown to exhibit dose-dependent cytotoxicity towards glioma cells and have shown synergistic activity with TMZ on U251 cells (93). In another study, AgNPs conjugated with anti-seizure drugs have been shown clinically to enhance amoeba-mediated host cell cytotoxicity (34). Also, silica is a commonly used metal nanoparticle for CNS drug delivery; it has been used together with lactoferrin to optimize the delivery and penetration of the BBB by receptor-mediated endocytosis. Its penetration ability is 1.6% greater when functionalized with PEG than glucose (94, 95). Other transitional metals like selenium, super magnetic iron oxide (SPIONs), and carbon dots have improved CNS delivery in silico (96, 97).

Animal studies have proven that selenium-loaded nanoparticles for CNS delivery have better results compared to standard therapy. Selenium-PLGA curcumin-loaded nanoparticle demonstrated cured memory deficiency in Alzheimer diseased–induced mice compared with curcumin used without nanoparticle delivery system. The binding and interaction of selenium NPs with amyloid-beta plaques were well visualized by fluorescence, thus could be used for both diagnosis and therapeutic purposes (96). An example is a pH-sensitive delivery of superparamagnetic iron oxide nanoparticle (SPION)–loaded doxorubicin to diagnose and treat glioblastoma. This study demonstrated optimal drug release at tumor pH environment in the treatment of glioblastoma compared to that of physiological pH (96).

Metal nanoparticles have been employed extensively in the diagnosis of CNS diseases. Recently carbon dots have shown superiority in biocompatibility, fluorescence, quantum yield, and uniform distribution compared to other bioimaging and therapeutic delivery techniques (98). Carbon dots’ photoluminescent properties make them excellent trackable imaging gene nanocarrier for transfecting plasmid DNA and siRNA into the CNS.

Though much has been said about metal NPs, their main functions are (i) to increase drug concentration at the luminal surface of BBB, (ii) to increase drug concentration in the brain, and (iii) enhance passive diffusion of the drug (99). These processes will establish a higher concentration of drug on the luminal side leading to rapid diffusion across the BBB resulting to higher accumulation in the brain. In addition, the surfaces of metallic NPs can easily be functionalized using polymers, charges, and antibodies to optimize delivery by targeting and optimal performance at the optimum pH (99).

### Direct Administration of Drugs into the Brain

#### Intracerebral Administration

One strategy for delivering drugs to the brain is to circumvent the problems associated with penetration of the BBB by direct injection of drugs into the brain. This approach is invasive, requiring a craniotomy in which a small hole is drilled in the head for intracerebroventricular (ICV) or intracerebral (IC) drug administration into the brain. An advantage of this approach is that a wide range of compounds and formulations can be considered for ICV or IC administration. Thus, both large- and small-molecule therapeutics can be delivered to achieve sustained release either alone or in various polymer formulations. Aside from the invasiveness of the craniotomy procedure and the implications this may have for long-term therapy, the biggest disadvantage of direct implantation of a drug into the CNS is related to the limited brain distribution of the drug. This phenomenon of limited tissue distribution is not restricted to large macromolecules.

#### Intrathecal Administration

Intrathecal administration has been re-examined as a means of circumventing the BBB delivery problems associated with large macromolecules such as proteins and peptides (100). Intrathecal administration involves the injection or infusion of drugs into the cerebrospinal fluid (CSF) that surrounds the spinal cord.

#### Nasal Administration

**Fig. 7:**
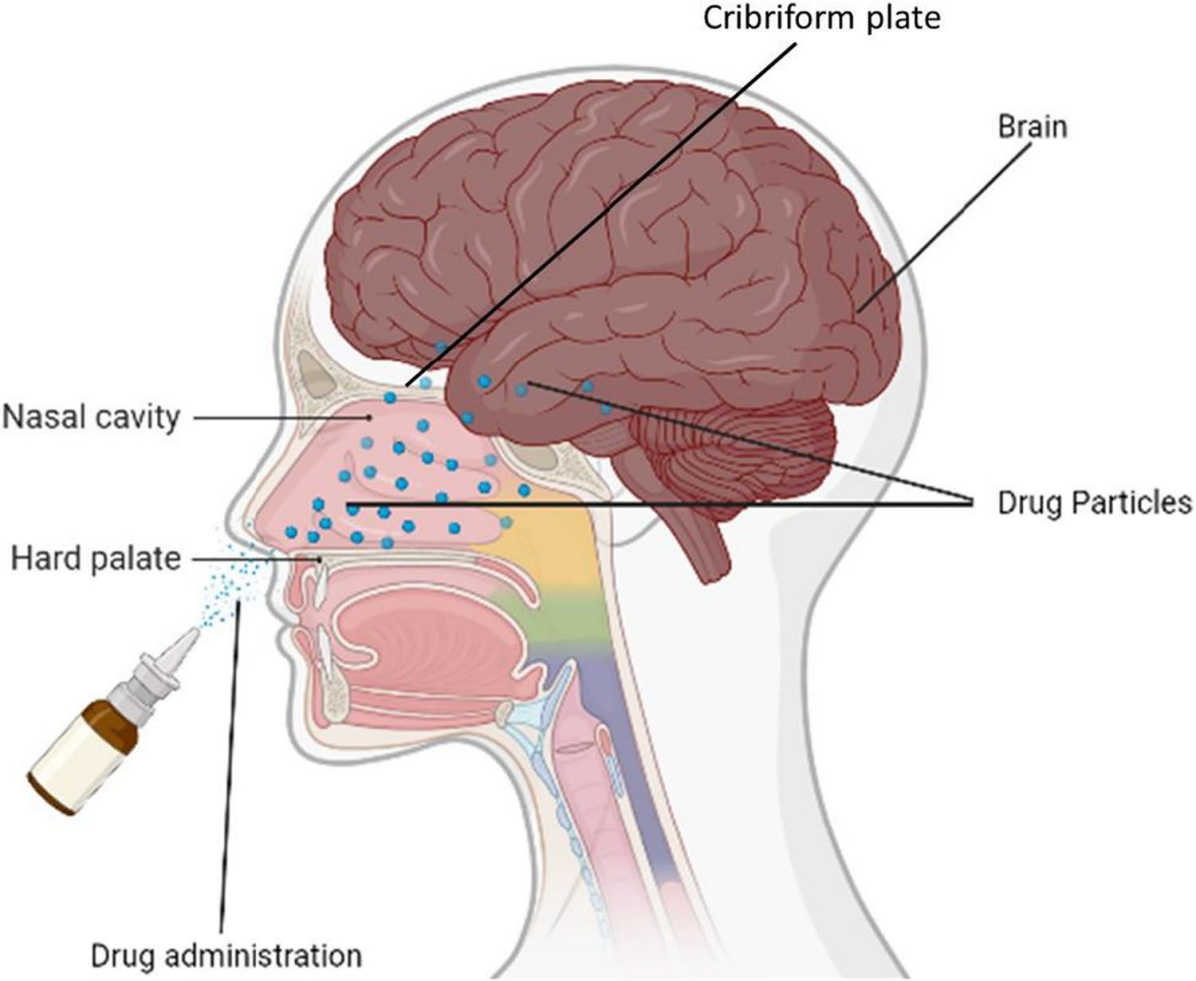
Intranasal drug delivery route into the CNS. Intranasal drug delivery into the brain via neuronal cells of the cribriform plate and tight junctions of the frontal lobe of the cerebral hemisphere

The nasal route has been utilized for the delivery of drugs for local diseases like nasal allergy and congestion. Recently, the attention of researchers has been drawn to the use of the nasal route for the administration of drugs to the brain due to its reliability, safety, non-invasiveness, and suitability (Fig. 7). One of the primary goals of most drug administration routes is to facilitate faster and higher levels of drug absorption into the systemic circulation of which the nasal route is not different (101). Bhattamisra and colleagues conducted a research study on rotigotine, a non-ergoline dopamine agonist formulated into a chitosan nanoparticle for delivery to the brain using the nasal route. The nanoparticles were formulated using the ionic gelation method, characterized by their optimal polydispersity index (PDI), zeta potential, size, and drug entrapment efficiency. Sprague Dawley rats with Parkinson’s disease were used to determine the pharmacological activity of rotigotine chitosan nanoparticles (RNPs) using the nose-brain delivery route. It was observed that the behavioral and biochemical testing of RNPs in Parkinson’s disease rats showed a reverse in catalepsy, akinesia, and restoration of swimming ability. RNPs improved brain targeting efficiency and dug bioavailability (102).

Wang and colleagues also developed rotigotine-loaded polymer micelles (ROT-PMs) thermosensitive gel as a delivery system to improve the solubility of drugs, increase residence and drug concentration into the brain for the treatment of Parkinson’s disease. The mean residence time of ROT-PM was extended via the nasal administration route compared to that of the intravenous route suggesting that ROT-PM has a potential application as a delivery system for nose-to-brain (103).

The research performed by Ahmad and colleagues showed the use of methoxy-poly(ethylene glycol) poly(D, L-lactide) copolymer (mPEG-PDLLA) and transactivator of transcription (TAT) functionalized polymeric micelles as delivery systems labeled with aggregation-caused quenching (ACQ) probes in nose-to-brain route of administration. The aggregation-caused quenching (ACQ) probe provided a fluorescent switch that tracked the release and identification of the nanocarriers produced. Through their findings, it was observed that TAT modification was able to reduce the retention of micelles in the nasal cavity resulting from improved absorption and distribution to the brain. Translocation of nose-to-brain was performed by *in vivo* imaging system (IVIS) live and ex vivo imaging (104).

### BBB Disruption

Under normal conditions, the complex tight junctions that form between the brain capillary endothelial cells restrict the paracellular diffusion of molecules and solutes in the BBB. Modification of the tight junctions, causing controlled and transient increases in the permeability properties of the brain capillaries, is another strategy that has been used to increase drug delivery to the brain. Methods for disrupting BBB integrity through the breakdown of tight junctions include the systemic administration of hyperosmotic solutions, vasoactive compounds such as bradykinin, and various alkylglycerols.

#### Osmotic Agents

Disruption of the BBB using osmotic agents has been extensively studied in both laboratory animals and clinically in the treatment of brain tumors (105, 106). In most cases, a hypertonic solution of an inert sugar, such as mannitol or arabinose, ranging from 1.4 to 1.8 M, is delivered into the cerebral circulation through bolus injection or short-term infusion into the carotid artery (107). The proposed cellular mechanism behind the osmotic disruption of the BBB involves the physical pulling apart/breaking of tight junctions due to the shrinkage of cerebral endothelial cells and expansion of the blood volume caused by the addition of the hyperosmotic agent.

As the disruption of the BBB is contingent on the presence of hyperosmotic agents in the blood, the BBB resumes its normal barrier functions within hours of returning the osmolarity of the blood to normal (108). During this period when the tight cellular junctions between the brain capillary endothelial cells have been compromised, paracellular diffusion of water-soluble drugs and solutes into the brain is enhanced.

Increased delivery of drugs to the brain following osmotic disruption of the BBB has been demonstrated in a variety of settings. Increases in small- and large-molecule delivery to the brain have been reported. The time course for disruption of BBB integrity and the subsequent return of the barrier function following osmotic disruption appears to be variable. Studies in rats suggested that the onset of BBB opening was rapid, with disruption not lasting more than 72 h after hyperosmotic mannitol administration (109). Likewise, return of normal BBB integrity was noted within minutes following cessation of the osmotic agent.

More recent studies in humans suggest that while disruption of BBB permeability in response to hyperosmotic mannitol was rapid, with increases in BBB permeability observed within 30 s to 2 min of the osmotic agent, the barrier properties were rapidly re-established within 20 min after the infusion was stopped (110). Osmotic disruption of the BBB has been used to increase the delivery of chemotherapeutic agents to the brain in the treatment of CNS tumors in rats (111). The clinical benefits of increased delivery of chemotherapeutic agents to the brain through osmotic disruption of the BBB have been demonstrated by the increased survival rates observed in patients with primary CNS lymphoma and malignant gliomas.

#### Bradykinin Analogs

The BBB can also be disrupted by pharmacological means. Several endogenous proinflammatory vasoactive agents, such as bradykinin, histamine, nitric oxide, and various leukotrienes, induce increases in BBB permeability in a concentration- and time-dependent manner (112–114). These vasoactive compounds are characteristically ultra-short-acting due to either rapid deactivation through metabolic processes.

#### Alkylglycerols

A relatively new approach for transient disruption of the BBB involves the systemic administration of various alkylglycerols. Reports show a reversible and concentration-dependent increase in BBB permeability to several anticancer and antibiotic agents (115, 116). The extent of BBB disruption varied from a twofold to a 200-fold increase in methotrexate, depending on the length of the alkyl group and the number of glycerols present in the structure.

### Non-invasive Physical Methods to Improve Delivery of Drugs to the Brain

Magnetic resonant–guided focused ultrasound (MRgFUS) seems to be the most advanced technology to facilitate drug to across the BBB (117). This is a unique recent approach for drug delivery across the BBB involving the reversible opening of the BBB using continuous exposures of short pulses of focused ultrasound (pFUS) to produce primary mechanical effects, with temperature elevations of only 4–5 °C (84). This is particularly possible with intravenous, commercially available ultrasound contrast agents (UCAs)—lipid- or protein-encased gas microbubbles that are 1–10 microns in diameter—to enable fine control over BBB. The microbubbles typically cluster near capillary walls when injected. The presence of pFUS exposures at low frequencies and pressure amplitudes instills stable cavitation and, therefore, localizes the pFUS effects to the endothelial cells. This significantly reduces the energy needed for BBB disruption, enabling low pressures that reduce the risk of heating the skull. Thus, stable cavitation of intravenous microbubbles induces transient, reversible BBB disruption (100).

**Fig. 8:**
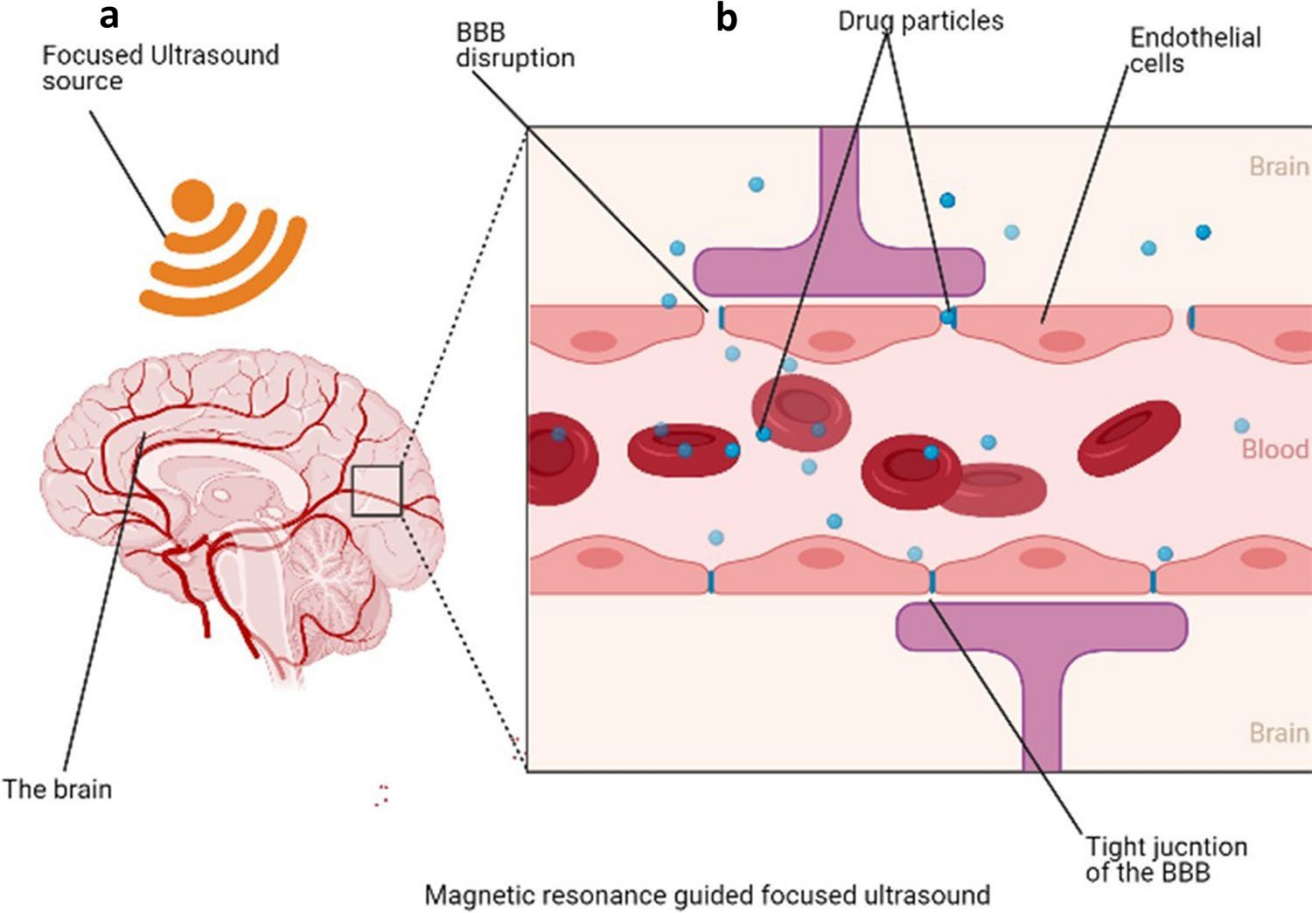
Magnetic resonance–guided focused ultrasound disruption of the BBB (**a**). Interruption of tight junctions by ultrasound waves for easy delivery of drug molecules into the brain (**b**)

A phase 1 clinical trial performed showed evidence of BBB disruption favoring the passage of drugs (Fig. 8). It increased the concentration of chemotherapeutic drugs in the sonicated brain tissues compared to the un-sonicated tissues to improve aggressive gliomas (117). Also, implants continue to be used and modified for sustained delivery in the CNS. The use of biodegradable wafers seems to be the future of postoperative optimized methods of overcoming the BBB for delivery. Studies have shown that modified wafers with unidirectional delivery improve postoperative outcome (118). Moreover, in a similar study done with a focused ultrasound technique, microbubbles were used to create cavitation to ease drug delivery to the CNS (119). In this same study, closed-loop cavitation operating at a frequency of 274.4 kHz was designed and used in normal and F98 glioma brain to demonstrate reliable and damage-free delivery of liposomal doxorubicin into the CNS resulting in increased concentration of drug delivered and improved tumor regression and survival in rat glioma (Fig. 8).

Most recently, the majority of studies have focused on the delivery of chemotherapeutic drugs and monoclonal antibodies, and facilitated cell-mediated delivery. In one study, the cellular entry following pFUS-mediated BBB disruption showed a five-fold increase in targeted natural killer cells within a brain tumor following intravenous administration of the cells (120). Indeed, this has potential in gene therapy for congenital disorders (e.g., lysosomal storage diseases), neurodegenerative diseases (e.g., Alzheimer’s disease and Parkinson’s disease), and brain tumors.

In a recent study by Vezina and colleagues, endothelial cell–cell interaction was decreased and fenestration upregulated after WNT/beta-catenin was activated to produce WNT antagonists indirectly and direct use of ICG-001 to block the WNT/beta-catenin signaling interactions (121). Regadenoson is an adenosine receptor agonist which has demonstrated a transient increase in the permeability of the BBB after intravenous administration of dextran and temozolomide in cancer patients (122). Another area of interest seems to be the use of bacterial hyaluronidase to enhance BBB permeability. This approach effectively uses *streptococcus agalactiae hyaluronidase* (HylB) to promote the penetration of *E. coli* M5 strain into the CNS of mouse models, indicating that activation of the HylB gene induced BBB opening in a dose-dependent manner (33).

Laser interstitial thermotherapy **(**LITT) is a non-invasive procedure that has recently been developed to induce BBB disruption through the administration of heat. LITT is a novel technique that allows for destroying tumor cells via laser ablative hyperthermia. It has various applications, such as the treatment of glioma, brain metastases, radiation necrosis, and epilepsy. It has been used as a safer alternative treatment option for patients in whom surgery was not possible and failed standard treatment options (99). LITT can destroy cell membranes, consequently leading to coagulative necrosis of the tumor. Meanwhile, cell membrane destruction also induces BBB/BBTB disruption, which may provide a chance for brain entry of anti-tumor agents.

To date, LITT has been successfully used for the treatment of a panel of tumors including, importantly, the LITT-induced thermal ablation region where the compromised BBB/BBTB occurs has been observed by following gadolinium-enhanced magnetic resonance imaging (MRI). Nonetheless, whether BBB/BBTB crossing of chemotherapeutic agents is enhanced following LITT application remains still unknown. It is expected that the disruption to the BBB will enhance drug access to target tissues in the treatment of CNS diseases. Current research is focusing on a combination technique for adjuvant chemotherapy or radiation following LITT and a combination of immunotherapy and LITT (123).

## CONCLUSION

The BBB is a dynamic assembly that helps protect the brain from adversity but stands as a major obstacle to drug delivery to the CNS. The progress achieved in drug delivery through various methods to overcome the BBB has shown significant success; however, a lot more has to be done including the use of multiple approaches such as targeting receptors using enhancers, inhibiting protein pumps, and using different routes for delivery. Nanoparticles are the most suitable form of drug loading for optimal delivery through targeting and enhancement as summarized in III. However, these methods are often accompanied by technical problems with devices, infection, and permanent damage to nearby brain tissue. Much more still needs to be done with many emerging neurological diseases to ensure safe and effective treatment options.
